# Uncontrollable bleeding after tooth extraction from asymptomatic mild hemophilia patients: two case reports

**DOI:** 10.1186/s12903-022-02074-9

**Published:** 2022-03-13

**Authors:** Guo Fan, Yi Shen, Yu Cai, Ji-hong Zhao, Yang Wu

**Affiliations:** 1 The State Key Laboratory Breeding Base of Basic Science of Stomatology (Hubei-MOST) and Key Laboratory of Oral Biomedicine Ministry of Education, School and Hospital of Stomatology, Wuhan University, 237 Luoyu Road, Wuhan, 430079 China; 2 The Department of Oral and Maxillofacial Surgery, School and Hospital of Stomatology, Wuhan University, Wuhan, China

**Keywords:** APTT, Mild hemophilia A, Persistent bleeding, Tooth extraction, Case reports

## Abstract

**Background:**

Uncontrollable bleeding after tooth extraction usually occurs in patients with coagulation diseases, including hemophilia, von Willebrand’s disease, vitamin K deficiency, platelet deficiency, and taking anticoagulant drugs. Hemophilia A is an X-linked recessive disorder caused by insufficiency of coagulation factor VIII. Mild hemophilia, defined by factor level between 0.05 and 0.40 IU/mL, is characterized by uncontrollable hemorrhage after trauma or invasive operations. Some mild hemophiliacs may remain undiagnosed until late adulthood. Therefore, surgical management of these patients may be relatively neglected. These case reports describe two uncontrollable bleeding patients with unknown mild hemophilia A after tooth extraction.

**Case presentation:**

This paper reports 2 cases of persistent bleeding after tooth extraction under local anesthesia which could not be completely stopped by routine treatments. Both of them denied prior illness and injury, allergies, anticoagulant medication history, systemic and family illness. The APTT and other coagulation screening tests of the two patients before surgery were normal. Finally, they were diagnosed with mild hemophilia A via coagulation factor assays. The patients acquired complete hemostasis by receiving coagulation factor supplement therapy in hematologic department.

**Conclusion:**

Mild hemophilia is marked by subclinical, asymptomatic and even normal coagulation test results. The purpose of these case reports is to bring dental professionals’ attention that APTT test alone cannot be used to exclude mild hemophilia, and provide reasonable evaluation and treatment procedures of bleeding patients after tooth extraction.

## Background

Hemophilia is an X-linked, recessive, bleeding disorder caused by deficiency of blood coagulation factor VIII. The incidence is estimated to be 24.6 in 10,000 males. It’s conventionally marked by occasional spontaneous bleeding or prolonged bleeding after minor trauma and abnormal coagulation test results. Coagulation screening tests are widely recommended to evaluate the risk of bleeding before oral and maxillofacial surgery [1, 2]. Platelet count, activated partial thromboplastin time (APTT) and prothrombin time (PT) are the major indicators. However, some mild bleeding disorders are often "subclinical" and the patients may experience increased bleeding only in the setting of trauma or surgical operations. Even worse, according to the World Federation of Hemophilia (WFH), whether the coagulation tests can accurately assess the coagulation status of mild hemophilia patients is still controversial [3].

Many studies have confirmed that these indicators cannot accurately intimate the coagulation function of patients due to some internal (patient's physical condition and some special coagulation diseases) or external (unregulated collection and processing of the sample) reasons [3, 4]. A patient with coagulation dysfunction might present "normal" laboratory examination results [3]. Similarly, an abnormal APTT result doesn't always indicate abnormal coagulation function because of internal or external influence.

Generally, most hemophilia patients are diagnosed preoperatively and tooth extractions are performed with a personalized hemostasis management plan. After performing a literature review, we found 2 cases of mild hemophilia after oral invasive surgery (tooth extraction [5] and electrosurgical resection of pericoronal flap [6]), while the two case reports lack preoperative APTT results. There are some patients with normal preoperative APTT results who had uncontrollable bleeding after surgeries (including tonsillectomy [7], ocular trauma [8], thyroidectomy [9]), and they were diagnosed with mild hemophilia A by factor assays eventually.

This paper reports 2 cases of persistent bleeding after tooth extraction which could not be completely stopped by routine treatments. Neither of them had a bleeding history. More specifically, their preoperative coagulation function tests showed "normal" laboratory examination results. Due to the poor effect of various hemostasis methods, we suspected that the patients had hemophilia and recommended a coagulation factor testing. Finally, they were diagnosed with mild hemophilia A via coagulation factor assays. Mild hemophilia is relatively uncommon compared with moderate to severe hemophilia which are characterized by spontaneous bleeding. It is generally believed that APTT can detect this coagulation disorder before surgery. However, we described two cases of mild hemophilia with a normal preoperative APTT result. The purpose of these case reports is to deepen the oral surgeon’s understanding of the preoperative coagulation screening tests, raise clinicians’ awareness of diagnostic tests and provide reasonable evaluation and treatment procedures for bleeding patients with potential mild hemophilia after tooth extraction.

## Case presentation

### Case 1

A healthy 29-year-old man came to our hospital and asked for the removal of his impacted wisdom teeth (#18 and #48) because of recurrent local inflammation. He mentioned that he went to the hospital a year ago because of bleeding gums and found abnormal blood coagulation (Table 1: 1 year ago). Despite that fact, he denied prior illness and injury, allergies, anticoagulant medication history, systemic and family illness.

**Table 1:** The coagulation test results of Case 1 at different time points

	1 year ago	Day 0	Day 5	Day 7
APTT (s)	**43.1** (20–38)	32.3 (20–38)	**53.5** (28–43.5)	**50.2 (**20–47.1)
PT (s)	11.3 (9–14)	9.4 (9–14)	13.1 (11–16)	14 (10–16.9)
TT (s)	17.3 (13–20)	16.6 (13–20)	15.5 (14–21)	14.9 (12.9–22.9)
INR	1.03 (0.8–1.3)	0.8 5(0.8–1.3)	1.01 (0.8–1.31)	1.12 (0.9–1.22)
FIB (g/L)			**4.53** (2–4)	3.25 (2–4)
FVIII:C%				**10** (70–150)

*APTT* activated partial thromboplastin time, *PT* prothrombin time, *TT* thrombin time, *INR* international normalized ratio, *FIB* fibrinogen, *FVIII* factor VIII, *s* seconds

The difference in reference value range (in the brackets) might be attributed to different coagulation tests devices among different medical institutions.

Coagulation screening tests were performed before tooth extractions. The international normalized ratio (INR), PT, APTT and platelet count were within the normal range (Table 1: Day 0). Under articaine infiltration anesthesia and lidocaine block anesthesia, two wisdom teeth were then routinely removed within 20 min. The wound was sutured with 2–0 unabsorbable sutures and compressed with sterile gauze as usual.

Several hours later, the patient developed continuous bleeding at the lower extraction site (#48). Because of the poor hemostasis effect of sterile gauze compression, we used a gelatin sponge (a tough and porous absorbable sponge-form material that makes the platelets adhere to the network and break down, thus leading to the release of thrombokinase [10]) to fill up the extraction site and sutured tightly to stop bleeding.

Second postoperative day, the patient had recurrent intermittent bleeding at both extraction sites. Each time the bleeding was relieved by sterile gauze compression. Fifth postoperative day, we performed coagulation screening tests again. The results showed that APTT and fibrinogen (FIB) were beyond the normal range (Table 1: Day 5). Then we began to consider whether there was a coagulation system disease, despite normal results obtained at day 0. Through a more detailed history inquiry, the patient recalled that his elder male cousin had "some problems in blood coagulation" before. We suggested he check the coagulation factor level at the hematology department.

Seventh postoperative day, severe persistent bleeding occurred again in both two sites. Neither sterile gauze compression nor the local application of hemostatic drugs could stop bleeding. We removed the original sutures under local anesthesia, cleaned the socket, filled the alveolus nest tightly with iodoform gauze, and then sutured tightly to achieve hemostasis.

The factor assays showed FVIII value decreased (Day 7, FVIII: 10%), while the level of FII, FV, FVII, FIX, FX, FXI, FXII were in the normal range (data not shown). The patient was diagnosed with mild hemophilia A.

At last, the patient acquired complete hemostasis after receiving twice coagulation factor supplement therapy (day 8 and 12, 400 IU of native plasma derived factor VIII). Iodoform gauze was safely removed 14 days after operation. Until the 28th day of postoperative follow-up, the patient had no hemorrhage again.

### Case 2

A 41-year-old man underwent extraction of his right mandibular second molar (#47) with severe periodontitis at a dental clinic, followed by continuous bleeding. He told the doctor that he had a physical examination a week ago and there were no abnormalities in his blood tests (data was missing) or other systemic diseases. Compressing with sterile gauze, extraction site suturing and injection of 2.0 IU Hemocaogulase Bothrops Atrox were used, but failed to achieve hemostasis. The APTT value was significantly increased to 66.4 s (normal, 28.0 to 43.5 s) in the blood coagulation tests (Table 2: Day 3).

**Table 2:** The coagulation test trsults of Case 2 at different time points

	Day 0	Day 3	Day 4	Day 5	Day 7
APTT (s)	Normal (data missing)	**66.4** (28–43.5)	**50.0** (20–38)	**58.5** (20–47.1)	
PT (s)		12.8 (11–16)	12.2 (9–14)	13.5 (10–16.9)	
TT (s)		14.7 (14–21)	17.2 (13–20)	14.1 (12.9–22.9)	
INR		0.98 (0.8–1.31)	1.11 (0.8–1.3)	1.02 (0.9–1.22)	
FIB (g/L)		**4.8** (2–4)		4 (2–4)	
FVIII:C%				**5** (70–150)	**3** (70–150)

*APTT* activated partial thromboplastin time, *PT* prothrombin time, *TT* thrombin time, *INR* international normalized ratio, *FIB* fibrinogen, *FVIII* factor VIII, *s* seconds

The difference in reference value range (in the brackets) might be attributed to different coagulation tests devices among different medical institutions.

When the patient came to our hospital (Day 4), we observed #47 alveolar cavity bleeding with swelling local gingival, and residual root of the lower right first molar (#46). Radiographs confirmed the #47 socket was empty. Coagulation function tests showed APTT prolonged to 50.0 s (normal 20–38 s) (Table 2: Day 4). The patient denied prior illness and injuries, allergies, anticoagulant medication history, systemic and family diseases.

Although the APTT value exceeded the normal range, it did not reach the alarm value of our hospital (APTT > 100.0 s, PT > 30.0 s). We suspected that the bleeding might be related to the inflammatory granulation tissue in the extraction socket. Therefore, we removed #46 residual root, gently cleaned both #46 and #47 sockets and sutured the wound under local anesthesia. Then the situation was similar as in Case 1. All routine methods could not stop bleeding until iodoform gauze was used to fill the extraction sockets.

Based on the experience of Case 1, we suspected this patient may be suffering from an asymptomatic coagulation dysfunction and suggested he had factor assays immediately.

The factor assays performed on days 5 and 7 diagnosed mild hemophilia A and the concentration of FVIII decreased continuously from 5 to 3% (Table 2: Day 5, Day 7). At last, the patient received FVIII supplement therapy to achieve a favorable prognosis without bleeding.

## Discussion and conclusions

Hemophilia is an X-linked congenital bleeding disorder characterized by a deficiency of coagulation factor VIII (FVIII), called hemophilia A (80–85% of all hemophilia cases), or factor IX (FIX), called hemophilia B (15–20% of all hemophilia cases) [3]. Hemophilia usually affects only males who inherit an affected maternal X chromosome, while female patients are rare. Estimated prevalence at birth is 24.6 cases per 100,000 males for all severities of hemophilia A, and the definite diagnosis of this disease is factor assays or other appropriate specific investigations, such as genetic testing [3].

According to the circulating level of FVIII, hemophilia A can be classified as mild (5–40 IU/dL or 5–40% of normal), moderate (1–5 IU/dL or 1–5% of normal), and severe (< 1 IU/dL or < 1% of normal), and the constitution ratio was mild 51%, moderate 25% and severe 21% respectively [3, 11]. Severe hemophilia usually has spontaneous bleeding into joints, muscles and soft tissues at an early age, and those with moderate hemophilia have occasional spontaneous bleeding or prolonged bleeding after minor trauma or surgery [11]. These two types of hemophilia are easy to diagnose according to medical history and clinical symptoms.

Patients with mild hemophilia usually do not have spontaneous bleeding, but only bleed abnormally after major surgery or trauma [11]. Therefore, they are probably unaware of hemophilia before tooth extraction, leading to uncontrollable postoperative bleeding.

Accurate diagnosis of hemophilia is essential to appropriate management and treatments. The APTT testing is conventionally used for assessing the contact factor pathway of blood coagulation, screening deficiencies in intrinsic pathway, monitoring unfractionated heparin (UFH) therapy, as well as screening lupus anticoagulant (LA) and assessing thrombosis risk [12]. It is considered to be a "global" coagulation test, which can indicate intrinsic and common pathway abnormalities or defects, including hemophilia [13]. Clinically APTT alone is generally considered to be an appropriate test for predicting individual bleeding risk during surgery [14].

However, studies found that APTT may not be able to exclude mild hemophilia. Luciá et al. [7] reported uncontrollable bleeding in a boy who underwent tonsillectomy at the age of 4, but no abnormalities were found in coagulation tests including bleeding time (BT), APTT and prothrombin, while the patient was diagnosed with mild hemophilia by coagulation factor assays at the age of 29. Hallet et al. [8] also reported a 12-year-old boy with an injured left eye. He had a normal preoperative APTT at 38 s (normal range 27–39 s), while his FVIII:C level was 25 IU/dL (mild hemophilia > 5–40 IU/dL; 5–40% of normal), and the boy was diagnosed with mild hemophilia eventually. As exhibited in our case report, the preoperative APTT test results were within the normal range in Cases 1 and 2, while both were eventually diagnosed with mild hemophilia by factor assays. The latest guidelines from WFH clearly state that APTT test results alone cannot be used to exclude the presence of mild hemophilia A or B, because APTT may be within the normal range in some cases of mild hemophilia [3].In addition, the results of APTT testing can be influenced by many factors, resulting in false positive or false negative results, and may lead to inappropriate precautionary measures or misjudgment, respectively [15] (Table 3).

**Table 3:** Interpretation of false APTT results

False negative results	Strenuous exercise, stress, inflammation, or pregnancy [16–18]
	The type of reagent used for the test [4]
	Compensatory of other higher level clotting factors [19]
	Inappropriate storage and handling conditions [3]
False positive results	FXII deficiency [20]
	Transient appearance of LAC [14, 21]
	The reduction of coagulation factors because of massive blood loss [22]
	Inappropriate storage and handling conditions [3]

*LAC* lupus anticoagulant

Above all, APTT test results alone cannot be used to exclude the presence of mild hemophilia. This disease will bring great challenges to oral surgeons due to its ambiguous clinical symptom and the unreliability of APTT test.

The diagnostic tests of hemophilia are factor assays and other specific investigations like genetic assessment. Oral surgeons should be acquainted with these technologies and give timely advice to patients with potential bleeding disorders.

Many guidelines suggest that it is not recommended to perform indiscriminate coagulation screening tests before surgery or other invasive procedures to predict postoperative bleeding in unselected patients [1]. A detailed preoperative inquiry of medication history and previous bleeding symptoms may be more useful than coagulation screening tests to predict the risk of bleeding after tooth extraction [14] (Table 4). This is particularly important for patients with potential hemophilia. Abnormal gingival bleeding, a common oral disease, may indicate coagulation dysfunction like hemophilia, platelet deficiency, von Willebrand’s disease, and the use of antithrombotic drugs, including fluindione, furosemide, amiodarone, paroxetine or ketoprofen [23].

**Table 4:** Detailed preoperative inquiry for suspicious patients

Preoperative inquiry	Contents
Previous bleeding symptoms	Continuous bleeding Muscle hematoma, joint bleeding, easy bruising, gingival bleeding Uncontrollable bleeding after undergoing surgical procedures
Family history	Tracking back to more than three generations, especially the male members
Systemic diseases	Liver disease: anemia, reduced hepatic synthesis of procoagulant factors, and increased fibrinolytic activity [24] Kidney disease: decreasing platelet function [25]
Medication history	Aspirin, heparin, clopidogrel and warfarin [26]

The National Institute for Health and Care Excellence (NICE) recommends tests of hemostasis for patients with a history of abnormal bleeding, anticoagulant medication or liver disease [27, 28]. Because abnormal results may indicate a significant underlying hemostatic dysfunction and it would also provide a useful baseline for the monitoring of factor VIII titer postoperatively [28]. Giuseppe et al. [14] reviewed 7606 patients and found that the risk of uncontrollable postoperative bleeding increased only when the APTT ratio > 1.3 (the reported therapeutic APTT range divided by the control value for the reagent, normal range 0.82–1.20), while a slight APTT prolongation (APTT ratio, 1.2–1.3) can be considered at "low risk of bleeding" irrespectively of the type of surgery.

We divide the patients into the following situations for reference according to preoperative inquiry and relevant blood tests:

1. Having a history of hemophilia: for these patients, a general surgical treatment plan from hematologists and comprehensive examination of coagulation function are necessary before tooth extraction.
2. Having a history of abnormal bleeding: regular coagulation screening tests should be performed before surgery. Routine surgical operations can be carried out on the patients with normal results; abnormal test results should be evaluated by hematologists before surgery.
3. Without bleeding history: for the patients without bleeding history, preoperative coagulation tests are not necessary. Routine treatments can be carried out, but bleeding disorders should be considered if the patients have uncontrollable bleeding like in the cases reported.

**Fig. 1:**
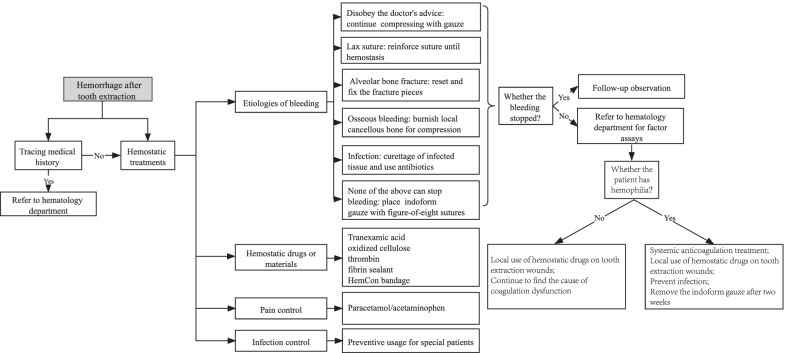
The procedures for bleeding patients after tooth extraction

Considering the relatively low incidence rate of hemophilia and high expense, it is not recommended to perform coagulation factor assays immediately for patients with abnormal bleeding after tooth extraction. Routine coagulation screening tests are recommended to rule out underlying severe disease. Meanwhile, surgeons should evaluate the etiologies of bleeding, and then deal with them respectively. Acute arterial hemorrhage after tooth extraction may be related to the rupture of large vessels in maxillofacial bone or soft tissue, or the less common but more critical vascular anomalies such as pseudoaneurysm or vascular malformation [29]. This acute bleeding can’t be managed effectively by local compression or packing of the socket. However, most patients with postoperative bleeding present with slow and continuous oozing which can be controlled by local interventions [30]. The recommended local treatment procedures are provided for reference (Fig. 1).

### Etiologies and managements of bleeding after tooth extraction

1. Failure to follow postoperative instructions: inadequate pressure of compressed gauze or eating too hot food may lead to blood oozing from the extraction site. It’s advisable to continue compressing with gauze until there is no more bleeding.
2. Lax suture: Slow bleeding can be seen from the wound. Reinforce suture under local anesthesia until there is no bleeding, observe for an hour with gauze compressed before discharging the patient.
3. Alveolar bone fracture: small fragments should be removed while the larger fracture pieces with sufficient blood supply should be retained by resetting and fixing.
4. Osseous bleeding: small-scale fractures of the alveolar bone due to excessive force during tooth extraction and the removal of bone resistance by the turbine may cause the nourishing blood vessels in the alveolar bone to rupture. Usually, it can be found during the operation. Aspirating to determine the bleeding site, a flat instrument (such as an elevator or periosteal raspatory) or Mitchell’s trimmer [31] can be used to burnish local cancellous bone to help compress for hemostasis in the area [32].
5. Infection: A large area of infection causes granulation tissue to form at the base of the socket, which may impair clotting and result in bleeding profusely [32]. The management of postoperative infection involves thorough irrigation of the wound site, curettage of infected granulation tissue and appropriate antibiotics [33, 34]. Incision and drainage of the abscess are required with abscess formation.

In addition to active local treatments, oral surgeons can use hemostatic drugs or materials around the tooth extraction socket, or systemically, to control hemorrhage, including tranexamic acid, oxidized cellulose, thrombin, fibrin sealant, gelatin sponge, and HemCon bandage, a special dental dressing [26, 35–37]. In addition, paracetamol/acetaminophen can be used for pain relief to alleviate the patient’s anxiety. It’s important to note that NSAID (non-steroidal anti-inflammatory drugs), including ibuprofen, are not recommended in hemophilia because it will aggravate the existing bleeding disorder by inducing platelet dysfunction [38]. For patients with severe trauma or bleeding caused by tooth extraction, antibiotics should be prescribed to prevent infection [39].

If the patient still bleeds after the above interventions, a packed iodoform gauze can be placed in the extraction socket with figure-of-eight sutures. At the same time, the patient should be sent to the department of hematology for detailed coagulation function examinations.

As a last-ditch emergency material, iodoform gauze can supply continuous and reliable physical pressure by fixing to adjacent soft tissue or tooth because of its relative incompressibility compared with conventional hemostatics, such as gelatin sponge or HemCon bandage. Moreover, after contacting with tissue fluid and blood, the iodoform can slowly decompose free iodine, which has a bactericidal effect. Iodoform has little irritation to tissues, and the effects of promoting granulation tissue regeneration will promote the healing of tooth extraction wounds [40]. This unabsorbable material needs to be removed 10–14 days after surgery. Iodoform gauze is usually used to stop bleeding and prevent infection in emergency management of high-energy shell fragment midface complex injuries [41] and hemostasis urgently in salvage treatment of hemorrhagic arteriovenous malformations in jaws [42].

In conclusion, tooth extraction is the most frequent invasive procedure in general population [43], and it is likely to be the most common surgical procedure required in people with hemophilia, especially those living in countries with restricted resources [44]. As an oral surgeon, it’s important to ensure the safety of the operation, perform timely treatment for bleeding patients and provide reasonable suggestions for patients with potential bleeding disorders.

Coagulation screening tests including APTT alone are not entirely reliable, especially for asymptomatic mild hemophilia patients. A detailed preoperative inquiry is more useful than unselective preoperative tests. We divided patients into the following three categories based on the results of coagulation tests and postoperative bleeding: (1) for the normal patients, routine tooth extraction treatment can be carried out; (2) for the bleeding patients with normal coagulation tests results, local hemostatic therapy can be performed; (3) hemophilia and other coagulation disorders should be considered if the results are abnormal and conventional treatments fail.

## Data Availability

Not applicable.

## Abbreviations

FVIII: Factor VIII
APTT: Activated partial thromboplastin time
PT: Prothrombin time
INR: International normalized ratio
FIB: Fibrinogen
TT: Thrombin time
s: Seconds
UFH: Unfractionated heparin
LA: Lupus anticoagulant
BT: Bleeding time
NSAID: Non-steroidal anti-inflammatory drugs
